# 

**Published:** 1917-05-19

**Authors:** 


### CONTENTS.

PAGE

**A Conflict of Opinion** ... 121

**The Criminal Law Amendment Bill** ... 122

**Hospital and Institutional News** ... 123
A New Director-General of the Navy Medical Service—The Ways of the War Office—A Curious Announcement—An Expert's Encouragement—Additional Hospital Staffs for France—The Effect of "Moderate Restriction" of Alcohol on the Public Health—War Charities to Benefit by Lotteries—An Institutional Wedding Gift—Quinn's Buildings, Waterloo Road—The King Edward VII. Hospital, Windsor—War Bonus for Infirmary Chaplains—Another Cancer Wing in London—The Clayton Hospital and Venereal Diseases—Giant at a Hospital—The National Baby Week—The Exposure of Food—What Lancashire Eats To-day—Beware of Rhubarb Leaves—Hospital "Gazettes" and the Supply of Paper—This Week's Drug Market—To Our Readers.

**Recuperative Hostels** ... 127

The Hankering after Euphemisms.

**The Service at Westminster Abbey** ... 128

**French Anti-Tuberculosis Measures** ... 129

**The Part and the Whole** ... 130

By Caduceus.

**Royal Victoria Infirmary, Newcastle** ... 131
Co-operation of City Corporation and Freemen.

**Parliament and the Profession** ... 132

PAGE

**From Across the Seas** ... 132
Municipal Control of Hospitals—Medical Charities at the Cape—News from New Zealand.

**The Matrons' and Sisters' Department** ... 133
Plain Words on Registration.—VI.
Round the Hospitals.
The Misleading and Betrayal of British Nurses. A Trap for the Prime Minister—Botany and Pretentious Humbug—Salaries and the Cape Hospital Board—An Amazing Manifesto.

**Some Problems of To-day** ... 135

IV. The Working Boy.

**Food in Wartime** ... 136

The Cost of War Rations.

**The Heating of Hospitals** ... 137

XII. The Nethern Asylum.

**Hospital and Institutional Results** ... 138

**A New Patent Electrolyser** ... 139

**Matrons' and Sisters' Appointments** ... 140

**Personalia** ... 140

**Passing Events and Topics** ... 140

**Gifts and Bequests** ... 140

**The Institutional Worker** ... (Suppl.) 1
Electro-Medical Apparatus.
Questions for May.
Questions Invited.

